# The readiness of the national health laboratory system in supporting care and treatment of HIV/AIDS in Tanzania

**DOI:** 10.1186/s12913-015-0923-z

**Published:** 2015-06-26

**Authors:** Leonard E.G. Mboera, Deus S. Ishengoma, Andrew M. Kilale, Isolide S. Massawe, Acleus S.M. Rutta, Gibson B. Kagaruki, Erasmus Kamugisha, Vito Baraka, Celine I. Mandara, Godlisten S. Materu, Stephen M. Magesa

**Affiliations:** National Institute for Medical Research, Headquarters, P.O. Box 9653, Dar es Salaam, Tanzania; National Institute for Medical Research, Tanga Research Centre, Tanga, Tanzania; National Institute for Medical Research, Muhimbili Research Centre, Dar es Salaam, Tanzania; National Institute for Medical Research, Tukuyu Research Centre, Tukuyu, Tanzania; Catholic University of Health and Allied Sciences, Mwanza, Tanzania; National Institute for Medical Research, Amani Research Centre, Muheza, Tanzania

**Keywords:** HIV/AIDS, Health laboratory system, Diagnosis, Tanzania

## Abstract

**Background:**

Strong health laboratory systems and networks capable of providing high quality services are critical components of the health system and play a key role in routine diagnosis, care, treatment and disease surveillance. This study aimed to assess the readiness of the national health laboratory system (NHLS) and its capacity to support care and treatment of HIV/AIDS in Tanzania.

**Methods:**

A documentary review was performed to assess the structure of the health system with reference to the status and capacity of the NHLS to support HIV diagnosis. Key informant interviews were also held with laboratory staff in all levels of the health care delivery system in four regions with different levels of HIV prevalence. Information sought included availability and utilization of laboratory guidelines, quality and the capacity of laboratories for diagnosis of HIV.

**Results:**

The findings indicate that a well-established NHLS was in place. However, the coordination of HIV laboratory services was found to be weak. Forty six respondents were interviewed. In most laboratories, guidelines for HIV diagnosis were available but health care providers were not aware of their availability. Utilization of the guidelines for HIV diagnosis was higher at national level than at the lower levels. The low level of awareness and utilization of guidelines was associated with inadequate training and supervision. There was a shortage of human resource, mostly affecting the primary health care level of the system and this was associated with inequity in employment and training opportunities. Laboratories in public health facilities were better staffed and had more qualified personnel than private-owned laboratories.

**Conclusion:**

Tanzania has a well established national health laboratory network sufficient to support HIV care and treatment services. However, laboratories at the primary health care level are constrained by inadequate resources and operate within a limited capacity. Improving the laboratory capacity in terms of number of qualified personnel, staff training on the national guidelines, laboratory diagnostic tools and coordination should be given a higher priority.

## Background

HIV/AIDS is one of the leading causes of morbidity and mortality among adult individuals globally [1]. The latest statistics indicate that about 35 million people worldwide were living with HIV in 2012 with an increase from previous years as more people are receiving the life-saving antiretroviral therapy; and about 70 % of these were in Sub-Saharan Africa (SSA) [1]. There were 2.3 (1.9–2.7) million new HIV infections globally, showing a 33 % decline in the number of new infections from 3.4 (3.1–3.7) million in 2001. During the same period, the number of AIDS deaths has declined with 1.6 (1.4–1.9) million deaths in 2012, down from 2.3 (2.1–2.6) million in 2005 [1].

In Tanzania, HIV/AIDS remains a major public health problem with enormous impact on human health and socio-economic development [2]. In an effort to combat the disease, Tanzania launched an HIV Care and Treatment Plan in October 2004 with the target of putting more than 400,000 AIDS patients on antiretroviral therapy (ART) by the end of 2008 [3]. The plan also aimed at tracking progression of the disease in 1.2 million HIV infected persons by the end of 2008. Recent analysis has shown that there has been an increase in new patients enrolled on ART from 2004 to 2009. However, the trend decreased from 2009–2012, most likely due to the decreased stigmatization, decreased incidence of the disease and increased awareness and linkage between counseling, testing and treatment [4].

Health laboratories form the backbone of health systems, as confirmation of aetiologies is critical for identifying specific diseases, guiding treatment, determining drug resistance and supporting disease surveillance [5–8]. However, in developing countries, the national health laboratory system (NHLS) faces multiple challenges related to inadequate budget and skilled human resources, poor infrastructure, lack of strong national policies and lack of understanding of the critical role that laboratories play in the accurate diagnosis and monitoring of patients suffering from high-burdens of diseases [3, 9–11]. This has resulted into inability to provide basic laboratory services to address the major diseases in the population [10, 12]. For HIV/AIDS, previous studies have shown that most of the laboratories in Tanzania had inadequacy in good laboratory practice (GLP), poor laboratory quality control for HIV testing and lacked internal and external quality control (QC) for the tests they were performing [13, 14]. Thus, major investments and reforms had to be done in order to support laboratory testing and clinical care of patients who would be assessed and eventually enrolled on ART [10].

National HIV/AIDS programmes are required to strengthen the capacity of laboratory services to be able to provide early testing for HIV and ensure appropriate care and treatment [15, 16]. In recent years, there has been a significant improvement of the NHLS in Tanzania in terms of infrastructure, equipment and human capacity to support diagnosis, care and treatment of people living with HIV/AIDS [10]. The national guidelines for voluntary counselling and testing (VCT) were developed in 2004 to provide guidance on HIV diagnosis and quality control (QC) and quality assurance (QA) procedures. Furthermore, a National HIV reference laboratory and zonal centres have been established with the mandate of conducting external quality control (EQC) and quality assurance (EQA) of HIV diagnostic services. The Ministry of health and Social Welfare (MoHSW), in collaboration with the USA Centers for Disease Control and Prevention (CDC) launched the Laboratory Logistics Systems to provide critical data on laboratory consumption to assist with tracking, managing, and resupplying laboratory materials [16]. Despite these developments, no systematic assessment has been conducted to assess the performance of the NHLS in Tanzania. This study was therefore conducted to assess the readiness of the NHLS in supporting care and treatment of HIV/AIDS in Tanzania.

## Methods

### Documentary review

Documentary review was performed to assess the structure of the health system in Tanzania with special reference to the status and the capacity of the NHLS to support HIV diagnosis, care, treatment, and disease surveillance. The national system for diagnosis of HIV and related infections was assessed in the context of the entire NHLS. This involved internet search for published papers, technical reports and other key documents. The search was performed through PubMed database and Google search engine. Key policy documents including guidelines, Strategic Plans, laboratory Standard Operating Procedures, legislations, unpublished monographs, and reports were also reviewed to describe the NHLS and the system for provision of HIV diagnostic services in Tanzania.

### Key informant interviews

#### Study area

These were conducted in 2013 and involved key informants at facility, district, zonal and national levels. Four regions of Iringa, Mtwara, Tabora, and Tanga in Tanzania were involved. The regions were randomly selected from the clusters with low, moderate and high HIV prevalence [2]. From each region, two districts (rural and urban) were selected whereby the district hosting the regional headquarter was purposely selected as an urban district while the rural district was selected randomly from a list of rural districts. The selected eight districts were Iringa Rural, Iringa Urban (Iringa), Masasi, Mtwara (Mtwara), Igunga, Tabora (Tabora), Muheza and Tanga (Tanga). In each district, a list of public and private health facilities (HFs) offering HIV testing and other related laboratory services by level (dispensaries, health centres, and hospitals) was developed. From each level, one health facility was randomly selected. However, where hospitals owned either privately or publically did not exist, one or two health centres were sampled to replace them. About six health facilities (public and private) were then selected from each district. The inclusion criteria of the health facilities (HFs) were: (i) availability of Care and Treatment Clinics (CTCs) and (ii) availability HIV laboratory diagnostic services. Facilities not providing HIV diagnostic services were excluded from the study.

#### Data collection

Key informant interviews were conducted with heads of the laboratories and or health facilities, and regional, zonal and national HIV laboratory managers. Data on Standard Operating Procedures (SOPs), laboratory guidelines, availability of quality control guidelines and their implementation and application, existence of national structure for laboratory management and other key guidelines related to quality assurance of HIV diagnostic services were also collected. Data on implementation of internal quality control and quality assurance (QC/QA) was collected at the health facilities as well as the involvement of the zonal laboratories in the external quality control and external quality assurance (EQC/EQA) programmes.

### Data analysis

Qualitative data from key informant interviews was entered into an excel spreadsheet by themes. The responses were weighted by tallying of those that were similar and later summarised in tables and texts. Analysis was done manually using thematic analysis framework which involved familiarization with the data, generating initial codes, searching for themes, reviewing themes, defining and naming themes and producing the report [17]. The triangulation method was used to establish the correlation of data in different themes. The different themes were then compared, discussed and agreed upon by members of the data analysis team. Findings from the documentary review were summarised and presented by themes showing the general structure of the health system, the NHLS and the system for HIV diagnosis in the country. The referral system for general health service delivery, general laboratory services and HIV diagnostics services is presented.

### Ethical considerations

This study received ethical approval from the Medical Research Coordination Committee of the Tanzania National Institute for Medical Research. Permissions to conduct the study in the regions and districts were obtained from the respective authorities. Written informed consent was obtained from each participant before the interview.

## Results

### Structure of health service delivery

The health system in Tanzania is organised in different levels. The dispensary (Level I) is the first formal health unit that serves between 6,000 and 10,000 people in one or more villages and provides basic outpatient services. The heath centre (Level II) serves >50,000 people and offers out and in-patient services, maternity care and laboratory services, and serves as the first referral for patients from dispensaries. A district hospital (Level III) serves as the second referral for the health centres and dispensaries, and provides out- and in-patient services. It also performs general surgical and obstetric operations. A regional hospital provides advanced referral services with high level of expertise compared to the district hospital. Higher levels of service delivery include the National Hospital (Muhimbili) and three Zonal referral hospitals (Bugando Medical Centre, Kilimanjaro Christian Medical Centre (KCMC) and Mbeya Referral Hospital) which provide services in their catchment areas of three or more regions in Eastern, Lake, Northern, and Southern Highland zones, respectively. These have highly qualified staff, specialized equipments and offer services similar to regional hospitals but at higher and specialized level. The health services at the dispensary, health centre and district hospital levels constitute primary health care services and are all managed by district councils. The primary care services and regional services operate under the Prime Minister’s Office, Regional Administration and Local Government as part of the government decentralization of services. The Ministry of Health and Social Welfare under the Central government provides overall health policy and guidelines.

### Laboratory system and HIV diagnosis

Provision of laboratory services follows a similar system as the general medical services. There is a National Health Laboratory System with a clear structure and laboratories are well established from the national level to health centres and dispensaries. There are estimated 6,217 laboratories which include the National Reference Laboratory (1), Zonal (4) and regional referral laboratories (23), district (90), health centres (577) and dispensary laboratories (5,522). Laboratories located at dispensaries and health centres are considered to be of the lowest level and should have one trained laboratory assistant with a minimum training of two years and a medical laboratory attendant. They have basic infrastructure including equipment and are capable of providing essential diagnostic services such as haemoglobin determination, blood slides for malaria, glucose measurement, sickle cell screening, urine analysis for glucose and proteins, sputum for diagnosis of tuberculosis (TB), stool for parasite detection and total/differential blood cell count.

The next level of the health laboratory system (level I) is located at the district hospital or other types of hospitals. These laboratories provide advanced services compared to lower levels with detailed investigations on samples such as urine, stool, blood/serum, cerebrospinal fluids, sputum and skin biopsies. The laboratories must have a minimum of six laboratory staff including two technologists/technicians, two laboratory assistants and two attendants under the leadership of the district laboratory technologist. Apart from supervising lower level laboratories, the district laboratory handles referred patients/specimens and keep records as well as managing health information generated by the laboratories in its catchment area and report that information to the MoHSW or other stakeholders (e.g. donors). Level II laboratories are located at regional hospitals and provide general and specialised laboratory services in haematology/blood transfusion, clinical chemistry and microbiology. The laboratory must have a minimum of 11 people including three specialised technologists (one in each of the three areas of haematology, clinical chemistry and microbiology), five general laboratory technologists/technicians and three attendants. Regional health laboratories are led by the regional laboratory technologists and they coordinate/implement laboratory quality assurance, supervision to district laboratories and provide referral services to lower levels.

The highest level laboratories (Level III) are located at the Muhimbili National Hospital and the three zonal referral hospitals of Bugando, KCMC and Mbeya. They are highly specialised with broad ranges of services and organised into five departments of haematology/blood transfusion, clinical chemistry, microbiology, parasitology and histopathology. These laboratories should have a minimum of 40 people including 15 specialist laboratory technologists, 15 general technologists/technicians and 10 medical attendants under the leadership of a zonal laboratory technologist. Zonal laboratories provide technical support to the catchment regions, coordinate/implement laboratory quality assurance system and other laboratory services as for the regional laboratories.

The National Health Laboratory Quality Assurance and Training Centre (NHL-QATC) was established in 2008 under the MoHSW and it is responsible for overseeing, coordinating and providing training on laboratory quality systems in both public and private health laboratories in the country. The NHL-QATC has been tasked to provide technical assistance and leadership in assuring quality systems integration and laboratory network in Tanzania, including coordination of external quality assessment schemes. The laboratory has advanced equipment and manpower for provision of quality assurance in order to ensure that all HIV testing services provided throughout the country are of the highest quality.

Overall, the national laboratory services and HIV diagnosis is coordinated by the Diagnostic Services Section of the Directorate of Hospitals of MoHSW. For HIV tests, laboratory diagnoses are carried out from zonal down to the dispensary level. Different HIV laboratory tests are performed at different levels with basic testing at lower levels and advanced tests performed at regional and Zonal laboratories (Table 1). Specialized laboratories use ELISA while lower health facilities (including health centres and dispensaries) use rapid diagnostic tests for both screening and diagnosis of HIV infections. Detailed guidelines of how to perform HIV testing and the diagnostic algorithm are provided by MoHSW. Similarly, other supportive diagnostic tests such as CD4 count, chemistry, haematology, infant diagnosis of HIV and detection of related infections, and the frequency of performing such tests have also been described.

**Table 1:** Tests and techniques for HIV and related diagnoses performed at different levels

Facility level	Test	Technique
Dispensary	Haemoglobin estimation	Haemoglobinometer, HaemoCue
	Sputum for AFB	ZN stain
	HIV screening	Rapid screening kits
Health Centre*	Total white cell count	Manual, using Turks Fluid
	Differential white cell count	Manual, using thin film
District Hospital**	Haemoglobin estimation	Hematology analyser
	Total and differential white blood cell, platelet and reticulocyte count	
	Blood indices	
	CD4 and CD8 count	
	Serum Albumin, bilirubin, SGOT, SGPT and Alkaline phosphatise	Flow cytometer
	Creatinine, Uric Acid, Cholesterol and Blood glucose	Chemistry analyser
	Serum electrolytes (Na^+^, K^+^, Cl^-^, Ca^2+^)	
	Total protein	
	Cryptococcus (CSF & Serum)	Negative stain, Latex antigen
	Bleeding time	Ivy
Regional Hospital***	HIV testing	ELISA
	Examination of CSF for yeast	Negative staining-India ink
	Reticulocytes and Eosinophil count	Vital staining
	G6PD screening test	Visual colorimetric
	Drug sensitivity testing	Disc diffusion
	Sputum for TB	ZN Stain
	AFB Microscopy	Light Emitting Diodes (LED) Microscopy
	Diabetes Mellitus Screening	Oral Glucose testing
	Glycosylated haemoglobin	Spectrophotometric
Zonal****	Hb types	Electrophoresis
	Serum proteins and C-reactive protein	Electrophoresis
	HIV testing	RNA & DNA PCR, Western blot
	TB culture	LJ, MIGT
	Blood culture	Bactec
	Lipid profile	Colorimeter, Spectrophotometer
	Lupus erythromatosus	LE cell bead extraction & staining

*Include all tests done at dispensary level; **Include all tests done at Health Centre level; ***Include all tests done at district level; ****Include all tests done at Regional level

Key: AFB = acid fast bacilli, Ca^2+^ = Calcium, Cl^-^ = Chlorine, CSF = cerebrospinal fluid, DNA = deoxyribonucleic acid, G6PD = glucose-6-phosphate dehydrogenase, Hb = Haemoglobin, K^+^ = Potassium, LJ = Lowenstein Jensen, MIGT = Mycobacterial Growth Indicator Tube, Na + = Sodium, PCR = polymerase chain reaction, RNA = Ribonucleic acid, SGOT = serum glutamic-oxaloacetic transaminase or aspartate aminotransaminase (AST), SGPT = serum glutamic-pyruvic transaminase or alanine aminotransferase (ALAT), TB = Tuberculosis, ZN = Ziehl–Neelsen stain

### National human resource for laboratory service

According to MoHSW statistics, by the end of 2012, there were 64,449 health care providers in the Tanzania Mainland. Of these, qualified laboratory staff (Laboratory Technologists/technician, Laboratory Scientists and Laboratory Assistants) accounted for only 3.2 % of the total staff. Of the professional human resource for health, there were 0.46 laboratory technologists per 10,000 population (Table 2). Further analysis of the laboratory staff in Tanzania indicates that there were more male laboratory staffs than females. However, the ratio differed between various laboratory cadres, with males accounting for the majority of the Health Laboratory Scientists and Laboratory technicians. About three quarters of the laboratory staffs were working with public-owned facilities. However, the majority of the Laboratory assistants were working with private (60.2 %) than public (39.7 %) health care facilities. Overall, most of the laboratory staffs were working in urban settings (59.7 %).

**Table 2:** Laboratory professional human resource by end of 2012

Cadre	Total number	Density per 10,000 population
Laboratory technologists	745	0.171
Assistant Laboratory Technologists	1,117	0.256
Health Laboratory Assistants	156	0.036
Health Laboratory Scientists	82	0.020
Medical Laboratory Technologists	27	0.006

Source: MoHSW (2013)

### Laboratory capacity for HIV diagnosis

Forty six key informants from public and private health facilities were interviewed including 22 (47.8 %) clinicians and 24 (52.2 %) laboratory personnel (Table 3). Despite low coverage at low levels, both public and private health laboratories of different levels were involved in the diagnosis of HIV.

**Table 3:** The number of respondents and types health facilities and their affiliation

Levels	Types and number (%) of health facilities		Total Number (%)
	Government	Private	
National/zonal laboratories	4 (9)		4 (9)
Regional/District Hospital laboratories	10 (22)	5 (11)	15 (33)
Health Centres	12 (26)	7 (15)	19 (41)
Dispensaries	6 (13)	2 (4)	8 (17)
Total	32 (70)	14 (30)	46 (100)

There was inconsistence regarding availability and human resource capacity for HIV diagnosis in terms of number and qualifications at the national, zonal/reference, regional and district levels. Whereas some respondents were of the opinion that human resource capacity was adequate, others felt that it was still a major setback for provision of high quality laboratory services. A respondent from the national level had these to say: *“We have adequate and competent staff.”* Yet, some respondents from the zonal laboratories said: *“Human resource is still a major problem. Employment and training are not equitably done”*

Laboratories in public health facilities had large number and more qualified personnel than private health facilities. The inadequacy of skilled laboratory personnel in private facilities was attributed to better salaries in the public compared to private sector which was described to offer low remuneration for laboratory staff. This was described to lead to increased involvement of non-professional laboratory staff in HIV testing. Most likely, such practice affected the quality of HIV laboratory services provided by the health facilities. Respondents also reported that the available laboratory personnel received training from the Ministry of Health and Social Welfare and the regional level. Interestingly, one respondent described HIV testing as a process that does not require specialized training: *“The capacity is good as HIV testing does not require specialized training”* (Respondent from a Health Centre).

Although there was a general agreement that laboratories had adequate equipment, some reported shortage of haematology analyser, calorimeter, chemistry analyser, viral load detection equipment and PCR machines. Some laboratories in higher level health facilities had shortage of key equipment such as refrigerators and haematology analyser. There was inadequate and untimely after-sale servicing and maintenance of the laboratory equipment. Even where servicing of the equipment was possible, it took a long time to get it done. This rendered the defective equipment unused when major services and repairs were required as testified by one of the respondents: *“It took eight months for the technical service providers to come and they never came on time****”*** (Key Informant, Regional Hospital). Although CD4 machines were available in most of the reference, zonal, regional and district laboratories, servicing of these machines was also hampered particularly when they ran short of cartridges. Respondents from lower health facilities (Health Centre and Dispensary) perceived the lack of equipment not a serious problem. This was because they were using HIV rapid tests for screening and diagnosis of HIV.

In most of the laboratories, availability of supplies was better though in some cases, delivery of the supplies was not done timely. This affected the quality of HIV laboratory diagnosis and the capacity to implement EQC and EQA at different levels. It was further noted that stock-out of some of the supplies and reagents such as those for haematology and biochemistry was not uncommon, especially reagents for CD4 count which was due to reliance on donor support and single source supply. Even when laboratory supplies were made available through the Medical Store Department (MSD), there was frequent mismatch between what was ordered and what was delivered. *“Sometimes, we do get wrong items due to mismatch between what is ordered and what is actually delivered*” (Respondent from a Health Centre).

### Availability, awareness, and utilization of guidelines for HIV diagnosis

Guidelines for HIV diagnosis were available in most of the laboratories. However, some respondents reported to have never seen such guidelines. To ensure quality HIV test results, service providers acquire knowledge through on job training and supervision. Such support services including supervision were reported to be rarely provided and few laboratory staff had opportunities for refresher training. One respondent from a Health Centre in Iringa had these to say: *“… though there is no documentation to show availability of guidelines, our laboratory operate in conformity to national guidelines such as adhering to national algorithm for HIV testing and take part in quality control”*.

The National Reference Laboratory was reported to use national, the World Health Organisation (WHO) and various supra national HIV laboratory guidelines for HIV testing. This was mainly due to the fact that they also took part in national and external quality control schemes. Study respondents had different levels of awareness on guidelines for HIV testing, but majority were not aware of the contents and the importance of the guidelines. Some respondents could not differentiate between SOPs and guidelines. Few respondents associated the low level of awareness to the involvement of few people who receive refresher training and supervision on laboratory procedures.

The study further observed that many respondents had mixed understanding about utilization of national laboratory guidelines for HIV diagnosis. For instance, non-adherence to guidelines was associated with the fact that the algorithm was difficult to follow. Utilization of guidelines was high at national level; but relatively low at the district, health centre and dispensary levels. The low utilization of guidelines at the lower facilities was associated with poor supervision and involvement of unqualified laboratory staff.

Most of the respondents agreed that the quality of HIV diagnostic services was good. However, a few mentioned some challenges to achieve better quality HIV and related laboratory diagnoses. These included heavy workload, insufficient laboratory space and lack of appropriate supplies and equipment for HIV screening. Lack of adequate laboratory space was described to affect confidentiality for provider initiated HIV testing and counselling (PITC). *“No privacy in laboratory services due to lack of enough space therefore counselling and testing are done in the same room…..”* (Respondent from a dispensary).

## Discussion

The findings of this study showed that Tanzania has a well established national health laboratory network sufficient to support care and treatment of HIV. However, it revealed some weaknesses in coordination and utilization of laboratory guidelines at different levels of the network. The laboratories also had shortage of skilled personnel that led to increased involvement of non-qualified health workers. Majority of the laboratories at the dispensary and health centre levels received inadequate supplies and had shortage of skilled personnel. Qualified and motivated human resources are essential for adequate health service provision, but the shortages have now reached critical levels especially in rural areas [18].

The current findings showed that human resource capacity at national, zonal/reference, regional and district levels was generally good. However, there were disparities on availability of qualified laboratory personnel within and between the studied districts, private and public health facilities; and between the different levels of the health delivery system. Generally, the laboratory staffs per 10,000 was quite low. Like in this study, in an analysis of the gaps in human resources, one study [18] reported a gap of laboratory technicians of 79 %. The gap was slightly higher in urban than rural districts. The findings that higher level laboratories had more qualified laboratory personnel as opposed to lower levels have also been documented in previous studies [12, 19]. In a review by Olmsted et al. [20], it was shown that despite considerable resources which have been invested in recent years to improve laboratory systems in resource-limited settings; human resources are inadequate, and the quality laboratory services is low at primary health care facilities. The disparity in the availability of qualified laboratory personnel between the levels can be associated with inequity in employment of new laboratory personnel and training opportunities among available staff. Furthermore, it was reported that more skilled laboratory personnel were deployed in some laboratories through donor funded projects and this raises great concern on issues of sustainability after completion of the projects. Our findings correspond well with those reported by other workers [13].

Within the country, there is a general shortage of human resources for health (HRH), compounded with mal-distribution that occurs at various levels. Relatively more health service staff are working in urban than in rural areas [21]. There is also an inequitable distribution of health care staff between public and private-not-for-profit sector facilities and between primary, secondary and tertiary facilities. Due to inadequate human resources especially at low level health facilities, it was shown that there was an increased involvement of unqualified/non-professional staff in provision of laboratory services for diagnosis of HIV/AIDS. Although this study did not attempt to document the driving factors for the increased involvement of non-professional laboratory staff in HIV testing, this may be a copping mechanism to address issues of shortage of skilled human resource in the health sector as previously reported [22]. Similarly, the extent of resultant effects of their involvement in HIV diagnosis was not documented.

In the study, it was reported that supplies including HIV diagnostic kits were mainly procured and distributed by the MSD [23] and most of laboratories were not satisfied with the delays in procurement and delivery procedures. Similar problems and others related to supply chain management of laboratory reagents, supplies and consumables have also been reported in the same districts (G. Kagaruki et al., unpubl.). While most laboratories had adequate and modern equipment, the capacity of laboratories down from the district level was not much a problem as they were using rapid tests for HIV screening. A general shortage of major laboratory equipment such as refrigerators and haematology machines was reported at higher level health facilities and this could have some effects on the quality of HIV diagnosis. However, lack of after-sale servicing of laboratory equipments which was associated with donor dependence could also significantly affect the quality of HIV laboratory services. A similar drawback to provision of quality HIV laboratory testing was the frequent interruption of supplies such as cartridges for CD4 machines which were reported to be used in most laboratories. However, even when laboratory supplies were made available through MSD, there was frequent mismatch between what was ordered and what was delivered as previously reported [23]. Delivery of mismatching products has been associated with increased stock-out of essential commodities which leads to poor quality of services provided by the health facilities, in terms of coverage, uptake and adherence; with a negative impact on service seeking behaviours [23–25].

Implementation of quality practices requires a systematic approach in particular, when the definition of a quality system is considered to be the comprehensive and coordinated efforts to meet quality objectives [26]. Our study showed that there was inadequate effort to ensure a systematic approach and resources availability for implementing critical activities to ensure quality laboratory services for HIV diagnosis. Although both healthcare and laboratory services were decentralized, the interaction between reference/regional level laboratories and counterparts at national level was not clear. Similarly, there was often little interaction between regional/district laboratories and their counterparts at the lower levels. This finding corresponds well with other reports which showed that laboratories in resource poor countries had limited capability and capacity for generating accurate and reliable data on disease diagnosis [27]. As also reported in this study, inadequate quality of HIV laboratory diagnosis at different levels leads to unreliable services and limited data for disease management and surveillance. Similar to other studies [27], we also did not document existence of a clear system that ensured appropriate dissemination of national guidelines to all levels, coordination of equipment maintenance and appropriate supply of repair facilities for laboratory equipment.

Our findings show that the national health laboratory policies/guidelines recommend HIV laboratory testing to be carried out from zonal down to the dispensary level. In Tanzania, a list of HIV and related laboratory tests required to support care and treatment at different levels of the health care system has been documented [10]. They include: HIV diagnostic assays using HIV rapid assays, ELISA, and PCR; disease staging and monitoring assays (CD4 count); drug safety assays (haematology and clinical chemistry); and tests for diagnosis of common and treatable sexually transmitted infections (syphilis) and opportunistic infections. Other tests include viral load and drug-resistance testing. However, our findings indicate that few dispensaries were involved in HIV testing and the service was mainly given to support PITC and prevention of mother to child transmission (PMTCT) services. Considering that a dispensary is strategically the closest health facility to majority of the community, lack of HIV diagnostic services at this level leaves a large segment of the population unattended. Although it has been noted that dispensaries have significant shortage of human resource, laboratory space and supplies, it will be convenient to deploy and provide laboratory HIV testing for VCT at dispensaries.

While national guidelines for HIV diagnosis were available in most of the laboratories, it was not clear on who was responsible to disseminate them and the mechanisms to ensure that each laboratory had and complied with the guidelines. A major concern was on the findings that some laboratory personnel had never seen the guidelines and others were unaware of the contents and the importance of the guidelines. This was mainly associated with poor supervision, lack of training and involvement of unqualified laboratory staff. The documented low quality of HIV laboratory diagnosis may also be associated with non-utilization of guidelines and SOPs at district, health centre and dispensary levels as previously shown by other studies [12, 19].

Interpretation of our study findings may be limited due to the design, selection of the study districts, duration of the study and interviews with key informants which were conducted by many interviewers. This may have affected the way the questions were asked leading to variations in responses, hence the interpretation of the findings. Selection of urban districts and some health facilities was done purposively. This may have allowed exclusion of districts and laboratories containing information which may have changed the current interpretation of the findings. Generalization of our findings is therefore appropriately limited to the study districts and regions.

## Conclusions

Although there was weak coordination, Tanzania has a well established national health laboratory network sufficient to support care and treatment of HIV services. Despite the important progress already achieved, most of the laboratories were resource constrained and operated within a limited capacity. Improving the laboratory capacity for HIV diagnosis remains an unavoidable priority.

## References

[1] UNAIDS (2012). Report on the Global AIDS Epidemic 2012.

[2] THMIS: Tanzania HIV/AIDS and Malaria Indicator Survey 2011-12. TACAIDS, ZAC, NBS, OCGS and ICF International, March 2013. http://dhsprogram.com/pubs/pdf/AIS11/AIS11.pdf.

[3] Somi G, Mattee M, Makene CL, Van Den HJ, Kilama B, Yahaya-Malima KI, Masako P, Sando D, Ndayongeje J, Rabiel B, Swai RO (2009). Three years of HIV/AIDS care and treatment services in Tanzania: achievements and challenges. Tanz J Hlth Res.

[4] MoHSW/WHO/IHI/NIMR: Ministry of Health and Social Welfare, WHO, Ifakara Health Institute & National Institute of Medical Research. *Midterm analytical Review of the Performance of the Health Sector Strategic Plan III 2009-2015*. Dar es Salaam, Tanzania, 2013. http://www.tzdpg.or.tz/fileadmin/documents/dpg_internal/dpg

[5] Panteghini M (2004). The future of laboratory medicine: Understanding the new pressures. Clin Biochem Rev.

[6] Wians FH (2009). Clinical laboratory tests: Which, why, and what do the results mean?. Lab Med.

[7] Gershy-Damet GM, Rotz P, Cross D (2010). The World Health Organization African region laboratory accreditation process: Improving the quality of laboratory systems in the African region. Am J Clin Pathol..

[8] Nkengasong JN (2010). A shifting paradigm in strengthening laboratory health systems for global health: acting now, acting collectively, but acting differently. Am J Clin Pathol.

[9] Gilson L, Magomi M, Mkangaa E (1995). The structural quality of Tanzanian primary health facilities. Bull Wld Health Org.

[10] Massambu C, Mwangi C (2009). The Tanzania experience: clinical laboratory testing harmonization and equipment standardization at different levels of a tiered health laboratory system. Am J Clin Pathol.

[11] Hiwotu TM, Ayana G, Mulugeta A (2014). Laboratory system strengthening and quality improvement in Ethiopia. Afr J Lab Med.

[12] Ishengoma DR, Rwegoshora RT, Mdira KY, Kamugisha ML, Anga EO, Bygbjerg IC (2009). Health laboratories in the Tanga region of Tanzania: the quality of diagnostic services for malaria and other communicable diseases. Ann Trop Med Parasitol.

[13] Mfinanga S, Mutayoba B, Mbogo G, Kimaro G, Kahwa A, Mhame P, Malecela M, Kitua A (2007). Quality of HIV/AIDS laboratory testing in Tanzania: a situation analysis. Tanz Hlth Res Bull.

[14] Mashauri FM, Siza JE, Temu MM, Mngara JT, Kishamawe C, Changalucha JM (2007). Assessment of quality assurance in HIV testing in health facilities in Lake Victoria zone, Tanzania. Tanz Hlth Res Bull.

[15] WHO (2008). Scale up of HIV-related Prevention, Diagnosis, Care and Treatment for Infants and Children: A Programming Framework.

[16] CDC: CDC in Tanzania: Factsheet, 2013. http://www.cdc.gov/globalhealth/countries/tanzania/pdf/tanzania-factsheet-final-june-2013.pdf

[17] Braun V, Clarke V (2006). Using thematic analysis in psychology. Qual Res Psychol.

[18] SIKIKA: Human Resources for Health in Tanzania: Deployment Tracking Survey. SIKIKA, November 2010.http://sikika.or.tz/wp-contents/upload/2013/human-resources-for-health-in-Tanzania.pdf.

[19] Ishengoma DRS, Derua YAR, Rwegoshora T, Tenu F, Massaga JJ, Mboera LEG, Magesa SM (2010). The performance of health laboratories and the quality of malaria diagnosis in six districts of Tanzania. Ann Trop Med Parasitol.

[20] Olmsted SS, Moore M, Meili RC, Duber HC (2010). Strengthening Laboratory Systems in Resource-Limited Settings. Am J Clin Pathol.

[21] MoHSW (2013). Human Resources for Health Country Profile 2012/2013.

[22] Munga MA, Kilima SP, Mutalemwa PP, Kisoka WJ, Malecela MN (2012). (2012) Experiences, opportunities and challenges of implementing task shifting in underserved remote settings: the case of Kongwa district, central Tanzania. BMC Int Hlth Human Rights.

[23] Kagaruki G, Kimaro H, Mboera L (2013). Factors Affecting Utilization of Evidence Based Health Information System for Effective Supply Chain of Essential Medicine in Tanzania: A Case Study from Mbeya Region. J Hlth Inform Devel Count.

[24] USAID: Strategies for Strengthening Laboratory Supply Chains. Deliver Project, Task Order 1.2008. http://www.who.int/hiv/amds/usaid_lab_supply2_2009.pdf.

[25] Kranzer K, Meghji J, Bandason T, Dauya E, Mungofa S (2014). Barriers to provider-initiated testing and counselling for children in a high HIV prevalence setting: a mixed methods study. PloS Medicine.

[26] MoHSW (2007). National Quality Assurance Framework.

[27] Martin R, Hearn T, Ridderhof J, Demby A (2005). Implementation of a quality systems approach for laboratory practice in resource-constrained countries. AIDS.

